# Effect of different types of supervised exercise programs on cardiorespiratory and muscular fitness, pain, fatigue, mental health and inflammatory and oxidative stress biomarkers in older patients with post-COVID-19 sequelae “EJerSA-COVID-19”: a randomized controlled trial

**DOI:** 10.1186/s12877-023-04544-3

**Published:** 2023-12-15

**Authors:** Eulogio Pleguezuelos, Sergio Sánchez-Nuño, Amin Del Carmen, Noemí Serra-Payá, Eva Moreno, Lorena Molina-Raya, Gemma Robleda, Marta Benet, Susana Santos-Ruiz, Ainoa Biurrun Garrido, Carmen Jerez-Molina, Marc Miravitlles, Mateu Serra-Prat, Xavier Viñals, Montserrat Girabent Farrés, Teresa Carbonell, Manuel V. Garnacho-Castaño

**Affiliations:** 1 Departamento de Medicina Física y Rehabilitación, Hospital de Mataró, Barcelona, Spain; 2 Departamento de Ciencias Experimentales y Sanitarias. Facultad de Ciencias de la Salud, Universitat Pompeu Fabra, Barcelona, Spain; 3 Grupo de Investigación DAFNiS (Dolor, Actividad Física, Nutrición y Salud), Campus Docent Sant Joan de Déu, Universitat de Barcelona, C/ Sant, C. de Sta. Benito Menni, 18-20, Sant Boi de Llobregat, 08830 Barcelona, Spain; 4 Servicio de Medicina Física y Rehabilitación, Hospital General de Hospitalet, L´Hospitalet de Llobregat, Barcelona, Spain; 5 Servicio de Neumología. Hospital Universitari Vall d’HebronVall d’Hebron Institut de Recerca (VHIR), Campus Hospital Barcelona, CIBER de Enfermedades Respiratorias (CIBERES) Barcelona, Barcelona, Spain; 6 Unidad de InvestigaciónConsorci Sanitari del Maresme, Mataró, Barcelona, Spain; 7 Departamento de Biología Celular, Fisiología e Inmunología, Universitat de Barcelona, Barcelona, Spain; 8 Facultad de Ciencias de la Salud, Universidad Internacional de Valencia (VIU), 46002 Valencia, Spain

**Keywords:** Rehabilitation programs, Endurance exercise, Resistance exercise, Cardiopulmonary exercise testing, Muscle strength assessment, Quality of life, Older people, SARS-CoV-2

## Abstract

**Background:**

Many patients with COVID-19 present the so-called post-acute sequelae of COVID-19 such as fatigue, post-stress discomfort, dyspnea, headache, pain mental impairment, incapacity to perform daily physical tasks ant exercise intolerance. This study aims to investigate the effects of different exercise programs on physical and mental fitness, physical condition and biomarkers of the immune system and oxidative stress in older patients with post-COVID-19 sequelae.

**Methods:**

The sample will be made up of 120 eligible participants, over the age of 60 years who have had COVID-19 disease and are survivors and present persistent COVID-19 symptomatology diagnosed by the corresponding physician. The participants will be randomly assigned to the experimental groups: supervised endurance group (SEG, *n* = 30), supervised strength group (SSG, *n* = 30), supervised concurrent group (SCG, *n* = 30), which will perform the corresponding exercise program 3 days a week compared to the control group (CG, *n* = 30), which will not carry out a supervised exercise program. The design of this project will include measurements of four relevant dimensions; 1) Cardiorespiratory fitness; 2) Muscle fitness; 3) Pain and mental health; and 4) Biomarkers of inflammation and oxidative stress.

**Conclusions:**

The results of this study will provide insights into the effects of different exercise programs on physical and mental fitness, physical condition and biomarkers of the immune system and oxidative stress in older patients with post-COVID-19 sequelae. These findings may be the basis for the formulation of health plans and rehabilitation programs that allow healthy aging and a reduction in the associated morbidity in patients with post-COVID-19 sequelae.

**Trial registration:**

NCT05848518. Registered on May 8, 2023.

**Supplementary Information:**

The online version contains supplementary material available at 10.1186/s12877-023-04544-3.

## Introduction

Coronavirus 2019 disease (COVID-19) was first identified in Wuhan, Hubei, China in December 2019 and is caused by the severe acute respiratory syndrome coronavirus 2 (SARS-CoV-2) [1]. The disease spread very rapidly to the rest of China and then throughout the world. In Spain, a state of alarm was declared on March 16, 2020 with the aim of managing the health care emergency produced by COVID-19. By August 9, 2022, 13,280,557 confirmed cases of COVID-19 and 111,339 deaths had been registered in Spain (https://www.sanidad.gob.es/profesionales/saludPublica/ccayes/alertasActual/nCov/documentos/Actualizacion_623_COVID-19.pdf). More than 100 million persons worldwide have been affected, with a hospitalization rate of 4.6 per 100,000 inhabitants. Fortunately, most of these patients remain alive [2, 3].

However, many patients with COVID-19 present the so-called post-acute sequelae of COVID-19. Between 10 and 20% of the patients with severe COVID-19 symptoms evolved with a phase of persistence of clinical manifestations such as fatigue, post-stress discomfort, dyspnea, headache, pain and many other neurocognitive conditions described such as mental impairment, and incapacity to perform daily physical tasks [4]. In addition, many patients present muscle weakness, intolerance to exercise [5] and a greater probability of developing stress, irritability, insomnia, confusion or frustration [6].

Consequently, many of these survivors of COVID-19 cannot perform their normal activities 60 days after hospital discharge [7], and approximately 60% of hospitalized patients present dyspnea, fatigue and muscle weakness as post-COVID-19 sequelae up to 7 months after hospitalization [8–10]. These post COVID-19 sequelae seem to be more evident in older persons with associated comorbidities, rapidly diminishing their quality of life [11] and, what is worse, increasing the risk of death in the older people compared with other age groups [12–14].

From a biological perspective, older people have shown to be more vulnerable to COVID-19, increasing the imbalance between reactive oxygen species and the mechanisms of antioxidant defense, exacerbating oxidative stress [15]. The dysregulation of immune function related to age can cause a more overt pathophysiological response to SARS-CoV-2 infection in older patients, which can accelerate the risk of biological aging, increasing oxidative stress, even after post-COVID-19 recovery [16].

Up to now, many pharmacological therapies have been used but few have demonstrated true effectiveness in post-COVID-19 survival and none, to date, has shown to reduce the sequelae of the disease, or specifically, the evolution of persistent symptoms even with global vaccination of the population [17].

There is overwhelming evidence that exercise produces benefits for health in the short, medium and long term, which prevents, delays, mitigates and even reverses many metabolic, pulmonary, cardiovascular, neurocognitive, inflammatory, rheumatic and musculoskeletal diseases, reducing fatigue and pain, increasing tolerance to exercise and daily life activities [18–23], and improving physical and mental health [24].

Although the need for effective scientific evidence-based exercise and rehabilitation programs in the period following COVID-19 is clear, the volume of patients requiring support and care will generate an unprecedented demand for medical care services worldwide. There are few studies on the benefits of exercise in the post-COVID-19 syndrome in older people. The last recommendations emphasize the need for physical activity adjusted to the symptoms and personalized exercise in post-COVID-19 recovery [25], with the aim of helping patients with post-COVID-19 sequelae achieve rapid recovery, improve their autonomy and, consequently, quality of life, especially in older persons.

Given the numbers of survivors suffering these post-COVID-19 sequelae, it is not only incentivizing but it is essential to produce knowledge to determine exercise schedules that are the most recommendable for improving the symptoms of the post-COVID-19 syndrome in relation to cardiorespiratory and muscular fitness (fatigue, muscle weakness), pain, mental health and biomarkers of inflammation and oxidative stress, taking into account the differences between older males and females.

### General, specific, transversal, partial and final objectives

#### General objective (GO)

Study the effects of different exercise programs on physical and mental fitness, physical condition and biomarkers of the immune system and oxidative stress in older patients with post-COVID-19 sequelae.

#### Specific objectives (SO)

Evaluate the effectiveness of different exercise programs in older patients with post-COVID-19 sequelae in relation to:

SO1. Muscle fitness; SO2. Cardiorespiratory fitness; SO3. Pain; SO4. Mental health; SO5. Biomarkers of the immune system; SO6. Biomarkers of oxidative stress.

#### Transversal objectives (TO)

TO1. Consider the perspective of sex and gender in all the SO.

#### Partial objectives (PO)

The partial objectives are designed according to the temporal needs of achieving the SO.

PO1. Recruit and select older patients with post-COVID-19 sequelae; PO2. Elaborate and supervise the exercise program according to the schedules established by the Department of Sports Medicine and Rehabilitation of the Hospital of Mataró; PO3. Perform evaluations of cardiorespiratory and muscle fitness, pain and mental health, and biomarkers of inflammation and oxidative stress prior to the initiation of the exercise program in older patients with post-COVID-19 sequelae; PO4. Collect the data and develop the database of the evaluations prior to the initiation of the exercise program; PO5. Implement the different exercise programs in older patients with post-COVID-19 sequelae; PO6. Perform the evaluations of cardiorespiratory and muscle fitness, pain and mental health and biomarkers of inflammation and oxidative stress in older patients with post-COVID-19 sequelae after completing the exercise program; PO7. Collect the data and develop the database of the final evaluations after the exercise program; PO8. Analyze and interpret the data.

#### Final objectives (FO)

FO1. Elaborate the corresponding reports; FO2. Report the knowledge generated to different informative media such as congresses, research meetings, scientific journals with impact, the media, etc.; FO3. Report and transfer knowledge of the results of the project to society: talks, activities in civic centers or centers for the older people.

## Methods

### Study design

**Fig. 1:**
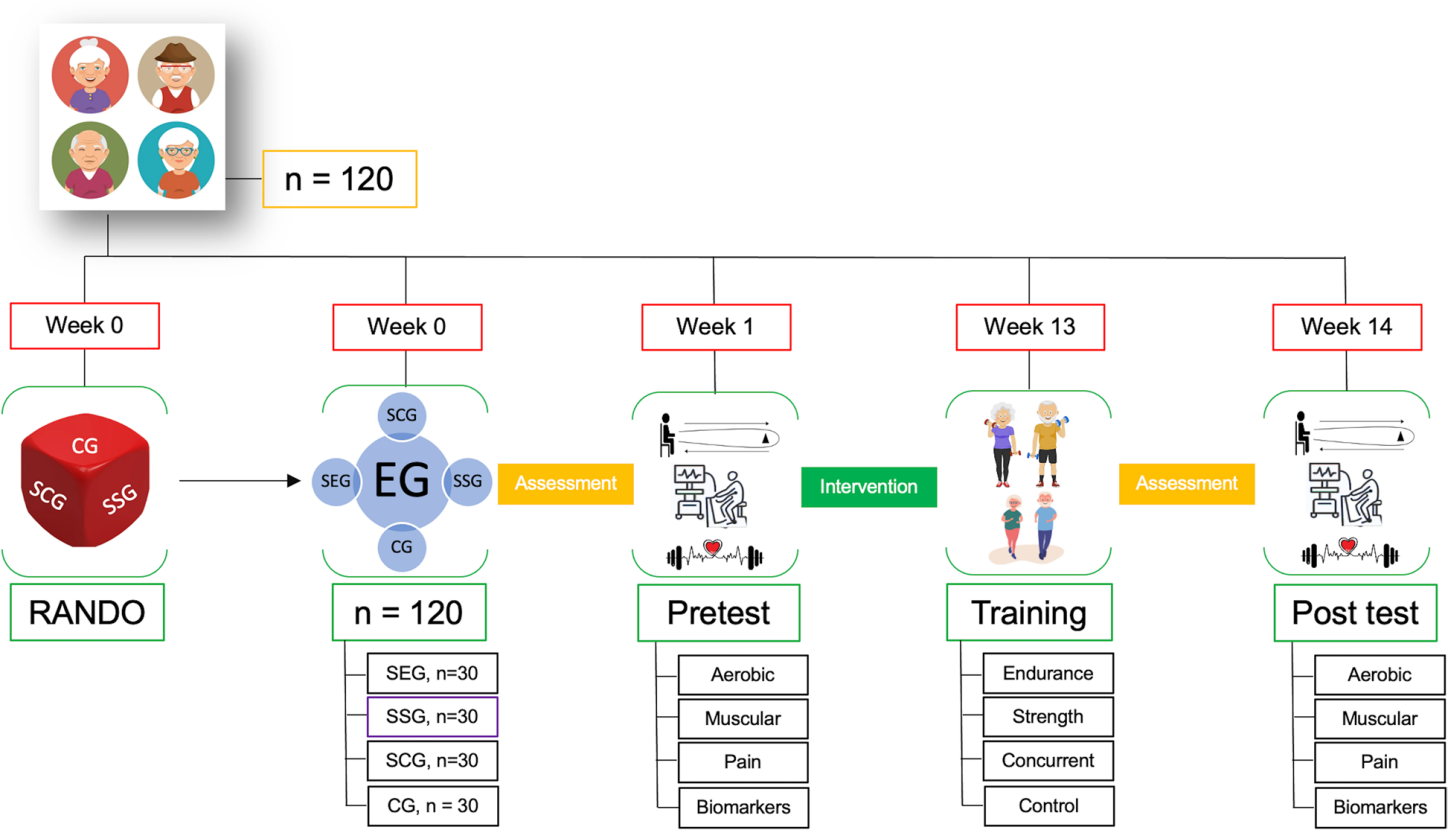
Study design. Abbreviations: CG = unsupervised control group; EG = experimental group; RANDO = randomization; SCG = supervised concurrent group; SEG = supervised endurance group; SEG = supervised strength group

The study design is shown in Fig. 1. This project will carry out a randomized controlled trial to determine what type of supervised exercise program is the most effective compared with a control group: supervised endurance group (SEG), supervised strength group (SSG), supervised concurrent group (strength and endurance) (SCG) and control group (CG). The Clinical Trials Registry is NCT05848518 (Last Update Posted: May 08, 2023). The protocol of study will be developed according to the Recommendations for Interventional Trials (SPIRIT) guidelines (Additional file 1) for randomized controlled trials [26].

The project will be divided into 3 subprojects and performed in coordination by three institutions as follows:

i) Subproject of the consolidated research group on pain, physical activity, nutrition and health (Acronym: DAFNiS) of the *Campus Docent Sant Joan de Déu. Universitat de Barcelona, Spain*. “Effects of a physical exercise program (aerobic, strength or combined/concurrent) on muscle fitness, pain and mental health in older patients with post-COVID-19 sequelae”.
ii) Subproject of the *Hospital de Mataró-Barcelona, Spain*: “Evaluation of cardiorespiratory and muscular fitness in older patients with post-COVID-19 sequelae”.
iii) Subproject of the *Departamento de Fisiología e Inmunología. Universidad de Barcelona, Spain*. “Evaluation of biomarkers of inflammation and oxidative stress in older patients with post-COVID-19 sequelae”.

### Participants and criteria of eligibility

The sample will be made up of 120 eligible participants, over the age of 60 years who have had COVID-19 disease and present persistent COVID-19 symptomatology with sequelae of fatigue, muscle weakness, pain, difficulty in performing daily life activities, etc. diagnosed by the corresponding physician.

Participants will be recruited from internal medicine services and patients with post-COVID-19 sequelae will be selected from the database of the hospital and primary care centers who have given their consent to be contacted for participation in the research project. The recruitment status of the participants will be ongoing at the time of manuscript submission.

Candidates will fulfill the following inclusion criteria:

- Persons of both sexes (and different expression of gender) older than 60 years of age.
- Molecular diagnosis (reverse transcription polymerase chain reaction [RT-PCR]) of infection by SARS-CoV-2.
- No history of physical diseases and no disease or disability that limits participation in the exercise program or precludes the corresponding measurement/evaluations.
- Be able to communicate without difficulty.
- Be able to understand the objectives of the project and provide written, informed consent.
- Not taking any medication that can affect normal performance of the exercise program and the evaluations. Specifically, patients who were treated with IL-6 receptor antagonists (example: tocilizumab) during the last month will be excluded. This type of medications will interfere with the exercise adaptations of the cardiorespiratory system.

The following exclusion criteria will apply:

- Having acute or terminal disease or any other disease that may affect the normal practice of a supervised exercise program and the corresponding evaluations.
- Consumption of any amount of alcohol or drugs during the study.
- Be performing any type of activity that may interfere in carrying out the supervised exercise program or the evaluations.
- Not completing the study once initiated or wishing to participate in the control group.

To calculate the sample size of this project a type 1 (α) error of 5% and a 95% confidence interval with a level of precision (d = 0.095) was established for the test of proportional hypotheses resulting in a sample size of *n* = 108 persons, increased by 10% (rounded up to *n* = 120) to compensate for possible dropouts along the study period.

To know the sample size more precisely in the hypothesis test of means it will be necessary to carry out a pilot study which will provide the size of effect and statistical power (0.80), determine the analysis to perform, obtain delta values in the units corresponding to the principal study variable as well as know the standard deviation. Since this cannot be carried out, the sample size described in the study by Udina and cols. (2021) [27] will be used, which established a slightly lower sample size (Total *n* = 33, subgroups of *n* = 20 and *n* = 13). Therefore, it is assumed that the theoretical sample calculation can be considered as correct given the above.

### Randomization, allocation concealment, and blinding

The eligible participants will be randomly assigned to the groups: SEG (*n* = 30), SSG (*n* = 30), SCG (*n* = 30), which will perform the corresponding exercise program 3 days a week compared to the CG (*n* = 30), which will not carry out a supervised exercise program. The method to generate the 1:1 allocation sequence to one of 4 experimental groups will be computer-generated random numbers (Research Randomizer). The assignment will be made using previously constructed balanced blocks until a total of 120 participants are assigned with the same number of participants per experimental group.

All participant assignment keys will be saved in a confidential file in the Campus Docent Sant Joan de Déu. An alphanumeric code will be assigned to each experimental group to guarantee masking. Once this process is completed, participants will be assigned to the corresponding experimental group. The alphanumeric code will be known by one of the researchers and will not be disclosed to the rest of the researchers until the analysis of the interventions is completed. Due to the characteristics of the intervention process in each experimental group, participants cannot be blinded to the exercise training programs. However, researchers and collaborators will be blinded to participant data during the baseline, intervention, and assessment processes. Blinding will be performed for all data.

The design of this project will include measurements of four relevant dimensions (Fig. 1): 1) Cardiorespiratory fitness; 2) Muscle fitness; 3) Pain and mental health; and 4) Biomarkers of inflammation and oxidative stress. In all cases, differences which may derive from variables of sex and gender will be considered.

### Program of supervised exercise

The intervention will consist in a program of supervised physical exercise performed 3 times a week for 12 weeks. For the planning and control of the exercise program the progressive individualized planning model will be applied based on the subjective perception of effort and cardiorespiratory parameters (heart rate and ventilatory threshold determined in the evaluation tests) of the participants. This program has been used by our research group in previous studies for improving cardiorespiratory and muscle fitness [28]. The basis of this periodization model establishes an initial period of individualization to obtain the personal parameters used for the training load.

Patients with post-COVID-19 sequelae will be randomly assigned to 3 programs of supervised exercise (Table 1).

**Table 1:** Summary of the characteristics of the supervised exercise programs

Session	EG	Methodology	WF	Dur (min)	NE	Set	Rep/T	Rest E/S	Intensity
Warm-up		General/ JM	3 s/w	5	1–3	1	5–15/20 s	15/60–120 s	HR: 40–50% RPE: 4–5
Workout	SET	Walking	3 s/w	50	1	1	1	––	HR to VT1:50–75% RPE: 4–8
	SST	Circuit training	3 s/w	50	10	1–4	5–25/60 s	15/60–120 s	HR to VT1:50–75% RPE: 4–8
	SCT	Walking + Circuit training	3 s/w	15 + 35	1 + 6–8	1 + 1–3	1 + 5–25/60 s	–– + 15/60–120 s	HR to VT1:50–75% RPE: 4–8
Cooling down		Stretching/ Relaxation	3 s/w	5	1–8	1	20–30 s	15/60–120 s	Resting HR RPE 1–3

*Abbreviations*: *Dur* Duration (minutes), *EG* Experimental group, *E/S* Exercise/set, *HR* Heart rate, *JM* Joint mobility, *NE* Number of exercises, *Rep/T* Repetitions/effort time, *RPE* Ratio of perceived exertion, *s/w* Sessions/weekly, *SCT* Supervised concurrent training, *SET* Supervised endurance training, *SST* Supervised strength training, *VT1* First ventilatory threshold, *WF* Weekly frequency

#### Program of endurance exercise

The program of supervised endurance exercise will consist in walking for 1 h 3 times a week. The intensity (step speed) will gradually be increased each week (approx. 5%) according to the perception of the participant. A pulsometer will be used to determine the safe range of heart rate at which the participant should perform.

#### Program of strength exercise

This will consist in performing a program of traditional strength 1 h 3 times a week. Circuit training methodology will be used (10 exercises) alternating muscle groups of the upper and lower extremities and trunk. The initial series be of 1 exercise, evolving to up to 3–4 along the program based on the individual adaptation of the individual. The intensity (load applied) will be gradually increased each week while the recovery times between each exercise activity will be reduced (approx. 5%) according to the perception of the participant. A pulsometer will be used to determine the safe range of heart rate at which the participant should perform.

#### Program of concurrent exercise (endurance and strength)

The participants will perform 1 h of concurrent exercise, combining walking (20 min) and the strength program for 40 min, maintaining the same instructions explained previously in each exercise program.

#### Control group

The control group will not carry out any supervised exercise program but will maintain their same habits of daily life activities.

Each supervised exercise session will include 10 min of general warm up and mobility exercises. For quality adequate control of the program and to stimulate the practice of collective exercise, the sessions will be performed in groups of 10–15 participants. Different strategies will be used to improve program adherence and fulfillment. The participants of the intervention groups of the project will be informed of the importance of attending all the sessions of supervised exercise as well as the evaluations made before and after the intervention period. In addition, a series of motivation strategies will be established for the participants of the intervention groups to increase or maintain the index of adherence to the exercise program including group dynamics to improve cohesion among the participants.

The CG will be supervised by a weekly phone call made by the members of the research group will the aim of knowing their health status and inform them of the evaluation periods and any other aspect that the participants consider relevant.

### Process of evaluation

To determine the effects of the exercise program, an initial evaluation will be made before beginning the exercise program to assess the initial status of the participants (Pre). This evaluation will be compared with the evaluation made at the end of the intervention program (Post) at 12 weeks.

#### Primary outcomes

##### Cardiorespiratory and muscle fitness

A series of functional physical condition used in the scientific community especially in older persons will be used [29]. At present, this series of tests has reference values for different age ranges from 60 to 94 years and cut-off points for each test associated with physical independence [30].

Aerobic capacity: this will be measured with the 6-min walk test, measuring the maximum distance (meters) walked in 6 min by the participants [29].

In addition, to complete the evaluation of cardiorespiratory fitness of the older adults, an incremental protocol on a cycle ergometer will be carried out using a respiratory gas analyzer. Depending on the characteristics of the participants, adapted standard protocols will be used [31–34].

Lower limb strength: this will be evaluated with the sit and stand test for 30 s. The number of times in 30 s the participant can completely stand up from a seated position with the back straight and the feet on the floor without pushing with the arms will be counted. A test will be performed to become familiarized with the test [29].

In addition, a test will be carried out to evaluate the explosive strength of the lower limbs by performing 3 countermovement jumps with 30 s of rest between each jump on a force platform (Musclelab, Ergotest Technology AS, Langesund, Norway) [35].

Upper limb strength: This will be evaluated with the bicep curl test made with a 2 kg weight for women and a 4 kg weight for men. The number of elbow curls made in 30 s with each upper limb will be counted [29].

Hand grip strength: This will be evaluated with the hand grip strength test. This test will be done with a digital dynamometer [36]. The participants will remain in the standard bipedal position throughout the test with the arm in full extension. Each participant will perform the test twice (alternatively with the two hands) with a 1-min rest period between each measurement.

Motor Agility/Dynamic balance: This will be evaluated with the 8-foot up and go test. The patient must get up from a chair, walk 8 feet towards and around a cone and return to the chair in the shortest time possible [29]. The best of two attempts will be registered.

In addition, a static balance test will be carried out on a force platform (Musclelab, Ergotest Technology AS, Langesund, Norway). Three attempts will be made in standing position with eyes open and 3 attempts with the eyes closed (if possible) with 1 min of rest between each attempt.

Flexibility/extensibility of the lower limbs: This will be measured with the “sit and reach test”. The patient will be seated with the leg extended and will slowly reach forward along the extended leg in an attempt to touch (or pass) the toes [29]. The number of centimeters lacking to reach the toes (negative score) or the number of centimeters that the toes are passed (positive score) will be registered. The best value of two attempts will be registered.

Flexibility/extensibility of the upper limbs: This will be evaluated with the “back scratch test”. This test provides a general measurement of the range of shoulder movement. It measures the distance between (or superposition of) the middle fingers behind the back [29]. It will be measured with both hands twice.

##### Evaluation of the biomarkers

The extractions will be made one week before initiation of the program and one week after completing the intervention.

Oxidative stress and antioxidant enzymes: Determination of oxidized lipids by the thiobarbituric acid reactive substances (TBARS) (fluorimetry) technique. Determination of oxidized proteins by the Advanced Oxidation Protein Products (AOPP) (colorimetry) technique. Determination of antioxidants by the total thiols technique (colorimetry). Determination of nitrites and nitrates (colorimetry).

Antioxidant Enzymes: Superoxide dismutase (SOD), catalase (CAT), glutathion peroxidase (GPx).

Inflammatory markers: Interleukin 6 and 10 (IL-6 and IL-10). Adiponectin. Tumor necrosis factor (TNF)-alpha. Serum IL-6 and IL-10 inflammatory markers and TNF-alpha will be determined with Luminex-100 IS (Integrated system: Luminex Corporation, Austin, TX, USA) system using the multiplex Linco High Sensitivity Human Cytokine Panel Lincoplex assay kit (Linco Research, Inc., MO, USA). Adiponectin will be determined with the Luminex-100 IS (Integrated system: Luminex Corporation, Austin, TX, USA) using the multiplex Linco Human Gut Hormone panel assay kit (Linco Research, Inc., MO, USA).

#### Secondary outcomes

##### Pain

Pain will be quantified with a pressure algometer + pupilometer in older patients with post-COVID-19 sequelae.

Pupilometer: The pupillary dilatation reflex (PDR) mode allows monitoring the variation of the pupil in real time during the application of a stimulus and during a maximum of 60 s.

The FLASH mode of the pupilometer, pupillary luminic reflex (PLR) allows evaluation of the reflex of pupilar constriction by a flash of light of 320 lumens during 1 s.

The pain pupillary index (PPI) mode uses standardized electrical stimulation that increases by 10 in 10 mA (initiating the stimulus in 10 mA up to 60 mA).

Parameters of the pupilometer: basal and maximum diameter of the pupil, percentage of variation of pupil diameter (in %), variation of the pupil in mm and intensity of reflex of dilatation, latency.

The pain threshold, subjective response to different painful stimuli, and objective response obtained after tetanic stimulation of the median nerve will be measured.

Pressure algometer: This commercial apparatus allows the application of a controlled and quantifiable nociceptive stimulus on a body surface.

Algometers apply a pressure stimulus that can be useful for applying a standardized painful stimulus, having a different nature from that applied with the pupilometer which is a stimulus of electric origin.

##### Mental health

Psychosocial variables will be evaluated using the Hospital Anxiety and Depression Scale (HADS) [37], which is made up of 7 items for anxiety and depression. Each item of the anxiety subscale (HADS-A) and the depression subscale (HADS-D) will be scored on a scale of 4 points from 0 (none) to 3 (much more). The highest scores indicate the highest levels of anxiety or depression.

##### Sociodemographic characteristics and quality of life

With the use of questionnaires age, sex, gender, marital status, educational level, socioeconomic level, family history, perception of health, presence of other diseases, medication consumption and the use of health services will be registered following the questionnaires of the National Health Survey as well as quality of life measured with Short Form Health Survey 36—SF36 [38].

### Data collection, management and analysis

An instruction manual for data collection and patient follow-up along the intervention process will be created. This manual will include the methodology of data collection and a detailed description of all the instruments used to obtain the study data. An initial protocol will be used for data entry, which will include specifications aimed at minimizing coding errors. All the technical personnel involved will undergo training sessions on the field work to harmonize data collection. Standardized protocols for quality control and data filtration will be developed. A centralized database will be designed for the data of the different participating centers. A central analytical plan will be developed, and the group coordinator of the project (*Campus Docent Sant Joan de Déu*) will provide data analysis and reports of study data in an agreed format. Only the principal investigator and the expert in research methodology and statistics will have information on the trial data until the end of the trial. Only anonymized data of the participants will be recorded.

An expert in research methodology and statistics will be in charge of designing the methodological focus and the statistical techniques that will be used to analyze the data obtained of each variable. Briefly, descriptive statistics will be calculated for the overall sample and between the experimental groups taking into consideration the gender perspective. Subsequently, the corresponding parametric or non-parametric analyzes will be carried out to establish the minimum intra- and inter-group changes of the variables analyzed. Missing cases will continue in the final analysis, however, they will provide less information and decrease the variance of the estimated effects to a lesser extent than the existing cases at each assessment stage.

An α value ≤ 0.05 will be considered to indicate statistical significance. The final analysis of the study will be fully described and differences between the enrolled experimental groups will be presented to determine the effectiveness of the exercise programs.

### Work plan, follow-up and evaluation

The project steering committee is made up of members of the DAFNiS group of the Campus Docent Sant Joan de Déu (Subproject 1: MVG-C, SS-N), the Mataró Hospital (Subproject 2: EP) and the Department of Physiology and Immunology of the University of Barcelona (Subproject 3: TC). This steering committee will be in charge of establishing the guidelines of the project through regular meetings either in person or by telephone, and by email.

To meet the objectives of the present project the activities indicated in the following diagram will be required. These activities are based on the objectives established, the subproject carried out, the investigators involved and the temporality of the same (Table 2).

**Table 2:**
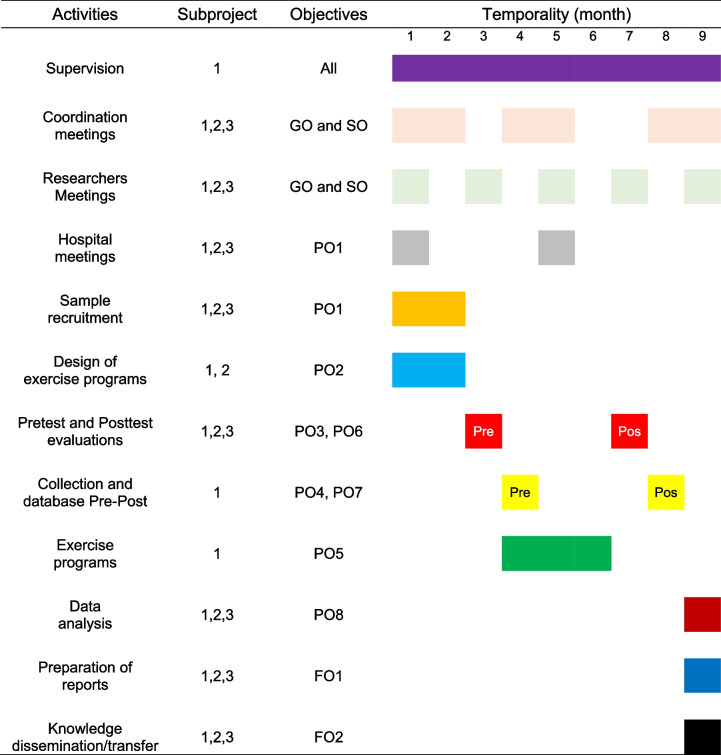
Work plan based on the activities, the subproject, the objectives and temporality

*Abbreviations*: *FO* Final objectives, *GO* General objective, *PO* Partial objectives, *SO* Specific objectives

In summary, two months are needed to select and recruit the sample and work in parallel on the design of the supervised exercise program. The intervention program will be of 3 months/12 weeks. The pre- and post- evaluations will be made at the beginning and after finalizing the intervention program of supervised exercise. Thereafter, data collection will be carried out as well as the creation of a database for the analysis and final interpretation of the data. Finally, the pertinent reports will be elaborated and the most relevant finding will be reported after the 9^th^ month in congresses, scientific journals, communication media, companies, etc.

As shown in Table 2, the research process will be followed with the goal of guaranteeing effective management of the subprojects, allowing exchange of information and efficient collaboration among the investigators of each subproject.

Along the project, frequent meetings will be held with the persons responsible for the other subprojects and with the investigators to assess the evolution and the incidences that may occur during participant recruitment, the pre- and post-evaluation process and during the exercise intervention program. In these meeting, all the team members will be informed of the status and evolution of the project and each subproject through reports, providing the correcting minutes of the meetings. On the other hand, the investigators will be asked to send the corresponding reports within adequate time periods to evaluate the evolution of the objectives.

In addition, it will be ensured that all the research activities adjust to the ethical norms applied in Spain and according to the ethical principles of the Declaration of Helsinki.

## Discussion

The present project aims to evaluate the effects of different exercise programs of physical and mental health, physical condition and biomarkers of the immune system and oxidative stress in older patients with post-COVID-19 sequelae.

COVID-19 has left its stamp on the world, affecting more than 100 million persons, and although the data are confounding, there have been around 15 million deaths according to the World Health Organization [39]. In Spain, up to August 9, 2022, 13,280,557 confirmed cases of COVID-19 and 111,339 deaths had been reported (https://www.sanidad.gob.es/profesionales/saludPublica/ccayes/alertasActual/nCov/documentos/Actualizacion_623_COVID-19.pdf).

Most of the persons presenting COVID-19 survive, but it is also true that many of the survivors present the so-called post-COVID-19 sequelae. Older persons are more susceptible to presenting these sequelae, especially those with associated comorbidities, subsequently affecting their quality of life [11] and increasing the risk of death [12–14].

Physical exercise has demonstrated to be one of the most beneficial regimens for short-, medium- and long-term health in all types of populations. It prevents, delays, mitigates and even reverses many metabolic, pulmonary, cardiovascular, neurocognitive, inflammatory, rheumatic and musculoskeletal diseases, reducing fatigue and pain and increasing tolerance to exercise and daily life activities [18–23]. In addition, it improves physical and mental health in older persons, especially in those who have had COVID-19 [24].

The EJerSA-COVID-19 project is clearly in line with the “Health, Demographic Change and Well-being” challenge of the Ministry of Health, given the implications that are expected to be obtained in the prevention of the consequences of ageing through physical exercise, and especially, in patients with post-COVID-19 sequelae. The interdisciplinary approach of this project aims to generate knowledge that can be transferred and applied to favor healthier aging in older persons, with special emphasis on health problems related to age and COVID-19 that have negative repercussions on cognitive, physical and mental status. As stated within the setting of this grant call, this project will cover 3 main axes:

1. Influence of physical activity that is beneficial to health on chronic non transmittable diseases (Post COVID-19)
2. Impact of physical exercise on psychological well-being.
3. Empowerment of the people: Active environments.

From this perspective, the development and undertaking of the present project will provide important support to the national and European policies on health and prevention of diseases by physical exercise beneficial for health. Although tertiary aspects of prevention are targeted (reduce or eliminate disease sequelae) in this project, the conclusions may be applicable to primary and secondary prevention.

Likewise, the focus of this project is aimed at two of the specific objectives established in the Strategy of Promotion and Prevention of Health of the Spanish National Health System:

1. “Promote active and healthy aging of the population over 50 years of age through integrated intervention in healthy lifestyles as well as in safe environments and behaviors in coordination with environments of health and community family”.
2. “Prevent functional impairment and promote health and emotional well-being in the population over 70 years of age by promoting the coordination of integrated interventions within the setting of health, and social and community services”.

Finally, it should be noted that the EJerSA-COVID-19 project also covers the objectives included in the Program of Investigation of the Plan of Physical Activity and Sports of the High Council of Sports, which already aims to “Promote investigation and studies in the field of physical activity and sport and the transfer of its results, especially in the association and impact of sports on health, school, physical activity adapted to handicapped persons and physical activity for older persons”.

Considering all of the above, we conclude by emphasizing the importance that this project will have with social and health care impact, and especially in older persons who have suffered COVID-19 and continue to have different physical and mental sequelae.

### Flexibility of the work plan

#### Limitations

The following limitations should be considered. The project will implement an intervention of 3 different programs of physical exercise in older patients with post-COVID-19 sequelae, and aims to determine which of the three programs is the most effective. The participants will be recruited through primary health care centers and hospitals, which makes the participant recruitment process difficult, and thus, the meetings of the supervisors of each center and with the medical personnel. The success of the process depends on medical recruitment, and it is difficult to assert that the complete estimated sample will carry out all the exercise programs. This is further complicated considering the possible study participant losses by persons who dropout for different types of reasons after having initiated the study (diseases, lack of motivation, post-COVD-19 symptomatology, among others).

On the other hand, during the intervention period, a series of strategies have been developed to promote adherence and participation with the aim of avoiding the greater than 10% dropout rate estimated for the study. Satisfactory adherence is considered with a minimum of ≥ 80% attendance to the scheduled exercise sessions. However, if the final expected sample size is not achieved in the initial recruitment or during the development of the exercise intervention programs and the dropout rate is higher than expected, a second recruitment and intervention phase could be implemented after the 5^th^ month of the project. If low recruitment continues and the estimated sample is lower than foreseen, the groups will be redistributed and patients with post-COVID-19 sequelae between 50–60 years of age will be included.

If with the previously described conditions we do not achieve a relevant sample based on the estimations made, a comparison will be performed between a group of older patients with post-COVID-19 sequelae who carry out a supervised plan with combined strength and aerobic exercises and a control group. In this case, it could not be investigated which exercise program is more effective, however, it could be determined if an exercise program combining aerobic stimuli of endurance with stimuli of strength is effective for improving persistent COVID-19 symptomatology is older people.

Finally, serious adverse events that affect the normal development of the intervention program will be immediately reported to the project steering committee, which will contact the ethics committee of the Mataró hospital (period less than 5 days) in order to preserve the protection and participant safety. The main serious adverse events will be diseases, infections, hospital admissions, death, etc.

## Conclusions

The results of this study will provide insights into the effects of different exercise programs on physical and mental fitness, physical condition and biomarkers of the immune system and oxidative stress in older patients with post-COVID-19 sequelae.

The promotion of investigation on physical activity and physical exercise as elements to promote health, and also including the perspective of gender, has not only scientific application (generation of scientific knowledge regarding the recovery of patients with diseases and/or older persons who have had COVID-19) but also social application in the everyday lives of this type of patients.

The foreseen results of this study will be applied in a sector of the population, that is older persons, who are those who have largely suffered the virulence of the disease, primarily affecting their physical and mental health. Any fundamental physical exercise therapy that reduces the devastating effects of persistent COVID-19 and improves physical and mental health will have sufficient social impact for use in programs of therapy and recovery in older patients with post-COVID-19 sequelae.

The findings of this study may be the basis for the formulation of health plans and rehabilitation programs that allow healthy aging and a reduction in the associated morbidity in patients with post-COVID-19 sequelae.

### Supplementary Information

**Additional file 1.** SPIRIT-Outcomes 2022 Checklist (for combined completion of SPIRIT 2013 and SPIRIT Outcomes 2022 items)^a^.

## Data Availability

All data of this study will be available upon reasonable request. Requests should be sent to the corresponding author.

## Abbreviations

AOPP: Advanced oxidation protein products
CAT: Catalase
CG: Control group
COVID-19: Coronavirus 2019 disease
DAFNiS group: Pain, physical activity, nutrition and health
EJerSA-COVID-19: Exercise for Health in COVID-19
GPx: Glutathione peroxidase
HADS: Hospital anxiety and depression scale
IL: Interleukin
PDR: Pupillary dilatation reflex
PLD: Pupillary luminic reflex
PPI: Pain pupillary index
SARS-CoV-2: Severe acute respiratory syndrome coronavirus 2
SCG: Supervised concurrent group
SEG: Supervised endurance group
SOD: Superoxide dismutase
SSG: Supervised strength group
TBARS: Thiobarbituric acid reactive substances
TNF-alpha: Tumor necrosis factor
